# Rifabutin Acts in Synergy and Is Bactericidal with Frontline Mycobacterium abscessus Antibiotics Clarithromycin and Tigecycline, Suggesting a Potent Treatment Combination

**DOI:** 10.1128/AAC.00283-18

**Published:** 2018-07-27

**Authors:** Mark Pryjma, Ján Burian, Charles J. Thompson

**Affiliations:** a Department of Microbiology and Immunology, University of British Columbia, Vancouver, British Columbia, Canada; b Centre for Tuberculosis Research, University of British Columbia, Vancouver, British Columbia, Canada

**Keywords:** Mycobacterium, Mycobacterium abscessus, antagonism, antibiotic combinations, clarithromycin, cystic fibrosis, drug regimens, rifabutin, synergism, tigecycline

## Abstract

Mycobacterium abscessus is a rapidly emerging mycobacterial pathogen causing dangerous pulmonary infections. Because these bacteria are intrinsically multidrug resistant, treatment options are limited and have questionable efficacy. The current treatment regimen relies on a combination of antibiotics, including clarithromycin paired with amikacin and either imipenem or cefoxitin. Tigecycline may be added when triple therapy is ineffective. We initially screened a library containing the majority of clinically available antibiotics for anti-M. abscessus activity. The screen identified rifabutin, which was then investigated for its interactions with M. abscessus antibiotics used in drug regimens. Combination of rifabutin with either clarithromycin or tigecycline generated synergistic anti-M. abscessus activity, dropping the rifabutin MIC below concentrations found in the lung. Importantly, these combinations generated bactericidal activity. The triple combination of clarithromycin, tigecycline, and rifabutin was also synergistic, and clinically relevant concentrations had a sterilizing effect on M. abscessus cultures. We suggest that combinations including rifabutin should be further investigated for treatment of M. abscessus pulmonary infections.

## INTRODUCTION

Recent epidemiological evidence shows that Mycobacterium abscessus is becoming a common hospital-acquired pathogen rather than an infrequent opportunistic environmental pathogen (1). A rapidly growing, nontuberculous mycobacterium with high levels of intrinsic antibiotic resistance, M. abscessus causes both local (soft tissue, surgical site, and lungs) and disseminated infections; it invades the lungs causing 18% of the nontuberculous mycobacterial infections in cystic fibrosis patients (2). Recent meta-analysis of patients with pulmonary infections has shown that only about one-third were able to clear M. abscessus infection after standard antibiotic treatment without surgery (3). Since none of the frontline antituberculosis drugs (including rifampin) have activity against M. abscessus, current treatment regimens are limited and must be improved. The American Thoracic Society has stated that “There are no drug regimens of proven or predictable efficacy for treatment of M. abscessus lung disease” (2).

Patients with M. abscessus infections are routinely treated with clarithromycin (CLR), along with two other antibiotics, usually amikacin (AMK) and either imipenem (IPM) or cefoxitin (FOX) (2, 4). Tigecycline (TGC) is sometimes used as a supplement to the triple antibiotic therapy when these antibiotics are ineffective. The efficacies of these antibiotics, especially in combinations, are limited by induction of resistance genes by CLR and by their pharmacodynamic properties. CLR activity is minimized by progressive induction of the rRNA methyltransferase *erm*(41) gene, which confers macrolide resistance (5, 6), and AMK activity is antagonized by CLR-induced resistance genes (7). Treatment of pulmonary M. abscessus infections is further complicated by the pharmacodynamic properties of TGC, IPM, FOX, and AMK, all of which have limited penetration into the lung (8).

The Sweet compound library (9) includes the majority of commercially available antibiotics (targeting DNA, RNA, protein, cell envelope synthesis, or essential metabolic conversions), as well as other physiologically active compounds. It was used in a screen to identify drugs with anti-M. abscessus activity. Rifabutin (RFB; a rifampin analog) was identified in the screen, and its activity against clinical isolates was verified. Since the current protocol for M. abscessus therapy employs combinations of CLR, AMK, FOX, IPM, and TGC, we investigated their interactions with RFB.

## RESULTS

### Rifabutin was the only rifampin analogue with activity against M. abscessus.

To identify antibiotics that targeted M. abscessus, we spotted the Sweet library onto lawns of M. abscessus ATCC 19977 and found compounds generating zones of clearance after 72 h. The high-throughput assay identified RFB as having anti-M. abscessus activity, which was confirmed by subsequent MIC determinations using M. abscessus ATCC 19977 and six independent clinical isolates (all strains showed a rough colony phenotype). All of the M. abscessus strains tested showed an RFB MIC of 6.3 mg/liter (Table 1). While the strains were sensitive to RFB, they had higher resistances to rifampin (MIC of 100 mg/liter), rifamycin SV (MIC of 25 to 50 mg/liter), and rifapentine (MIC of ≥50 mg/liter) (Table 1); this is consistent with previous reports (10, 11). The RFB MIC we determined in Mueller Hinton II (MHII) medium (6.3 mg/liter) confirmed a recent, independent study (11) showing that RFB's MIC was 3 mg/liter in 7H9 medium and 6 mg/liter in MHII medium. It is difficult to know which of these media best predicts the *in vivo* MIC.

**TABLE 1:** Susceptibility of rifampin and derivatives against M. abscessus

Strain^*b*^	Median MIC (mg/liter)^*a*^			
	Rifampin	Rifamycin SV	Rifapentine	Rifabutin
ATCC 19977	>100	25	50	6.3
Strain 1	>100	50	100	6.3
Strain 2	>100	50	100	6.3
Strain 3	>100	50	100	6.3
Strain 4	>100	25	50	6.3
Strain 5	>100	25	100	6.3
Strain 6	>100	50	>100	6.3

a Values are the medians of three experiments.

b All strains had rough colonial phenotypes.

### Synergies of rifabutin with macrolides and tigecycline.

To explore interactions between RFB and antibiotics used to treat M. abscessus infections (CLR, AMK, TGC, IPM, and FOX), growth inhibition was measured using checkerboard assays (12, 13). Checkerboard assays determine the fractional inhibitory concentration index (FICI), which defines the synergy between the compounds (FICI ≤ 0.75) (14–17). We examined the combinations using M. abscessus strain ATCC 19977, as well as our six clinical isolates.

RFB activity was not synergistic with AMK, IPM, or FOX (data not shown) but showed synergy with both CLR and TGC. The combinations of RFB with either CLR or TGC were synergistic against all seven strains (Table 2; representative plots of checkerboard results are shown in Fig. S1 in the supplemental material). Importantly, either TGC or CLR reduced the MIC of RFB at least 4-fold (except for strain 3, which showed a 2-fold change when paired with TGC); this lowered the *in vitro* MIC of RFB to concentrations that are found in the lung (∼2 mg/liter) (18). Treatments with macrolides other than CLR have been reported for pulmonary infections. These include azithromycin (AZM) and roxithromycin (ROX). RFB was also synergistic with AZM (FICI = 0.5) and ROX (FICI = 0.375) (Table 3; representative plots of the checkerboard are shown in Fig. S1 in the supplemental material).

**TABLE 2:** MICs of rifabutin in combination with clarithromycin and tigecycline

Strain	Median MIC (mg/liter)^*a*^									
	Rifabutin + clarithromycin					Rifabutin + tigecycline				
	Alone		In combination			Alone		In combination		
	RFB	CLR	RFB	CLR	FICI	RFB	TGC	RFB	TGC	FICI
ATCC 19977	6.3	0.2	1.6	0.05	0.5	6.3	0.8	1.6	0.4	0.75
Strain 1	6.3	0.4	1.6	0.1	0.5	6.3	1.6	1.6	0.4	0.5
Strain 2	6.3	0.2	1.6	0.05	0.5	6.3	0.8	1.6	0.4	0.75
Strain 3	6.3	0.4	1.6	0.2	0.75	6.3	0.8	3.1	0.2	0.75
Strain 4	6.3	0.4	1.6	0.2	0.75	6.3	0.8	1.6	0.2	0.5
Strain 5	6.3	0.4	1.6	0.1	0.5	6.3	0.8	1.6	0.2	0.5
Strain 6	6.3	0.4	0.8	0.2	0.63	6.3	1.6	1.6	0.8	0.75

a RFB, rifabutin; CLR, clarithromycin; TGC, tigecycline. Values are the medians of three experiments.

**TABLE 3:** MICs of rifabutin in combination with macrolides

Agent	Median MIC (mg/liter), macrolide + rifabutin^*a*^				
	Alone		In combination		
	MAC	RFB	MAC	RFB	FICI
AZM	3.1	6.3	0.8	1.6	0.5
ROX	3.1	6.3	0.4	1.6	0.38

a MAC, macrolide; RFB, rifabutin; AZM, azithromycin; ROX, roxithromycin. Values are the medians of three experiments.

**FIG 1:**
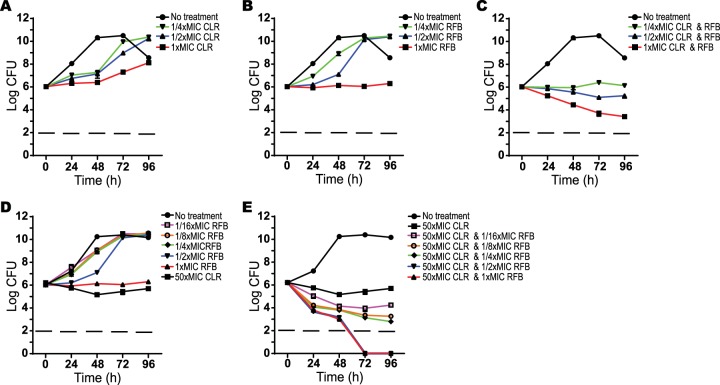
Effect of rifabutin and clarithromycin combinations on M. abscessus viability. The following antibiotics were added to M. abscessus ATCC 19977 cultures: clarithromycin at 1× (0.4 mg/liter), 1/2×, or 1/4× MIC (A); rifabutin at 1× (6.3 mg/liter), 1/2×, or 1/4× MIC (B); or a combination of clarithromycin and rifabutin at 1×, 1/2×, or 1/4× MIC of each antibiotic (C). The effect of rifabutin at higher clarithromycin concentrations was tested by incubating M. abscessus with rifabutin alone at 1× to 1/16× MIC (D) or in combination with 50× MIC (20 mg/liter) of clarithromycin (E). CFU were determined at 24-h intervals after antibiotic addition. Data points are the means from three replicates with standard deviations presented as error bars.

To build on the checkerboard assays that measure growth inhibition, the effect of RFB paired with CLR, AZM, or ROX on M. abscessus was examined using CFU analyses which measures viability (Fig. 1 and see Fig. S2 in the supplemental material). In all cases, the combinations noticeably improved the extent of growth inhibition, including bactericidal activity. None of these antibiotics alone caused decreases in CFU, and they only inhibited growth at 1× MIC. However, in combination, RFB at 1/4× MIC, along with 1/4× MICs of macrolides (CLR, AZM, or ROX) was sufficient to prevent growth of M. abscessus over 96 h. Combinations of RFB and any macrolide, both at 1× MIC, were bactericidal; moderate bactericidal activity was also observed using 1/2× MIC combinations (Fig. 1C and see Fig. S2C and F in the supplemental material).

Since macrolides accumulate in lung tissue at concentrations above their *in vitro* MIC (8), we also tested RFB in combination with higher CLR concentrations. Kill curves were generated by pairing various RFB concentrations (at or below the MIC for M. abscessus) with a clinically relevant concentration of CLR (50× MIC; 20 mg/liter). Even when CLR was added at 50× MIC, it showed no bactericidal activity (Fig. 1D). However, bactericidal effects were generated when 50× MIC of CLR was paired with RFB as low as 1/16× MIC (0.4 mg/liter) (Fig. 1E). CLR paired with RFB at 1/2× and 1× MIC showed CFU decreases to below the detection limit after 72 h.

**FIG 2:**
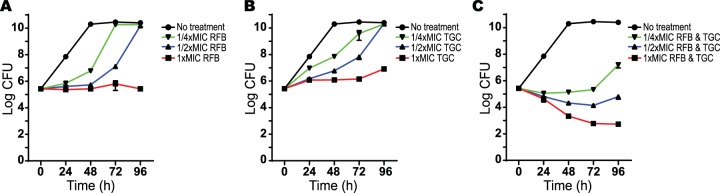
Effect of rifabutin and tigecycline combination on M. abscessus viability. The following antibiotics were added to M. abscessus ATCC 19977 cultures: rifabutin at 1× (6.3 mg/liter), 1/2×, or 1/4× MIC (A); tigecycline at 1× (0.8 mg/liter), 1/2×, or 1/4× MIC (B); or a combination of rifabutin and tigecycline at 1×, 1/2×, or 1/4× MIC of each antibiotic (C). CFU were determined at 24-h intervals after antibiotic addition. Data points are the means from three replicates with standard deviations presented as error bars.

The same kill curve analyses were done to analyze RFB and TGC interactions. RFB (1× to 1/4× MIC) or TGC (1× to 1/4× MIC) alone had no bactericidal effects (Fig. 2A and B). The combination of RFB and TGC at 1× or 1/2× MIC had bactericidal activity (Fig. 2C).

### Synergy between clarithromycin and tigecycline.

**FIG 3:**
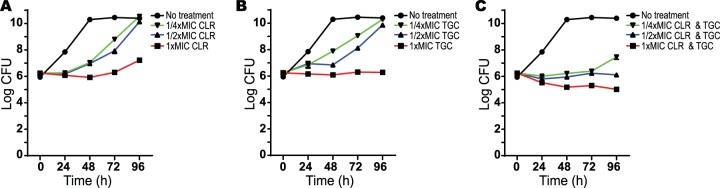
Effect of clarithromycin and tigecycline combinations on M. abscessus viability. The following antibiotics were added to M. abscessus ATCC 19977 cultures: clarithromycin at 1× (0.4 mg/liter), 1/2×, or 1/4× MIC (A); tigecycline at 1× (0.8 mg/liter), 1/2×, or 1/4× MIC (B); or a combination of clarithromycin and tigecycline at 1×, 1/2×, or 1/4× MIC of both antibiotics (C). CFU were determined at 24-h intervals after antibiotic addition. Data points are the means from three replicates with standard deviations presented as error bars.

Synergy of TGC and CLR has been previously reported and the combination is suggested for clinical use (19, 20). Their synergy in preventing growth was confirmed in our checkerboard assays (data not shown). To determine whether this combination also had bactericidal effects, M. abscessus was incubated with 1×, 1/2×, and 1/4× MICs of CLR and TGC, alone or in combination, and assessed for viability (CFU). In contrast to the combination of CLR and RFB (Fig. 1) or TGC and RFB (Fig. 2), the combination of TGC and CLR at 1× MIC only slightly reduced the CFU of M. abscessus (Fig. 3). Together, these data show that RFB enhances the bactericidal effects within all of these combinations.

### Rifabutin has potent synergy in triple combination with clarithromycin and tigecycline.

We had observed synergy or enhanced activity of RFB in combination with CLR or TGC. Three-dimensional (3D) checkerboard analyses were carried out to investigate whether a triple combination of CLR, TGC, and RFB could have greater inhibitory effects than double combinations. A 3D checkerboard was used to assess growth inhibition in the presence of all three antibiotics, with each drug in the combination assayed in a series of concentrations (see Fig. S3 in the supplemental material). The FICI was calculated as the lowest FICI value in each experiment using the median value of three independent experiments. Our results show that the combination of RFB, CLR, and TGC was synergistic with an FICI of 0.375; MIC values in triple combinations were lower than MIC values of any combination of two antibiotics (Table 4). 3D checkerboard analyses of the combination against six M. abscessus clinical strains were also performed. All strains showed a synergistic FICI of ≤0.625 (Table 4). This demonstrated that the triple combination was synergistic in all of the clinical strains. The complex assembly of data generated in each 3D checkerboard was visualized as a 3D surface (isobologram). Isobolograms of M. abscessus ATCC 19977 exposed to RFB, TGC, and CLR at different concentrations showed areas of concavity which indicate synergistic interactions (see Fig. S4 in the supplemental material).

**TABLE 4:** 3D checkerboard results for a combination of rifabutin, clarithromycin, and tigecycline

Strain	Median MIC (mg/liter)^*a*^						
	Alone			In combination			
	RFB	CLR	TGC	RFB	CLR	TGC	FICI
ATCC 19977	6.3	0.2	0.8	0.8	0.025	0.1	0.375
Strain 1	6.3	0.4	1.6	0.8	0.05	0.2	0.375
Strain 2	6.3	0.2	0.8	0.8	0.05	0.2	0.625
Strain 3	6.3	0.4	0.8	0.8	0.05	0.2	0.5
Strain 4	6.3	0.4	0.8	0.8	0.05	0.2	0.5
Strain 5	6.3	0.4	0.8	0.8	0.1	0.2	0.625
Strain 6	6.3	0.4	1.6	0.8	0.1	0.2	0.5

a RFB, rifabutin; CLR, clarithromycin; TGC, tigecycline. Values are the medians of three experiments.

**FIG 4:**
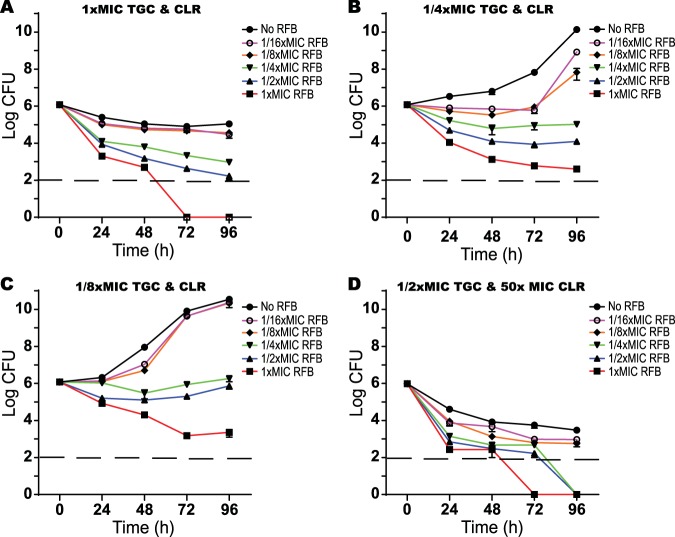
Effect of rifabutin, clarithromycin, and tigecycline combinations on M. abscessus viability. Rifabutin was added to M. abscessus cultures at 1× (6.3 mg/liter), 1/2×, 1/4×, 1/8×, or 1/16× MIC in combinations of clarithromycin and tigecycline at 1× MICs of tigecycline (0.8 mg/liter) and clarithromycin (0.4 mg/liter) (A), 1/4× MICs of tigecycline and clarithromycin (B), 1/8× MICs of tigecycline and clarithromycin (C), or lung *C*_max_ tigecycline (1/2× MIC) and clarithromycin (50× MIC) (D). CFU were determined at 24-h intervals after antibiotic addition. The dashed line represents the limit of detection. Data points are the means from three replicates with standard deviations presented as error bars.

Since the combination of RFB combined with either CLR or TGC had bactericidal activity against M. abscessus, the triple combination was similarly analyzed. M. abscessus was grown in the presence of 1×, 1/4×, or 1/8× MIC of TGC and CLR, along with various concentrations of RFB (1× to 1/16× MIC), and the viability was assayed by CFU (Fig. 4A, B, and C). At 1× MICs of CLR and TGC, M. abscessus viability was marginally reduced. However, addition of as low as 1/4× MIC of RFB (1.6 mg/liter) caused a large reduction in CFU, with 1× MIC of RFB yielding culture sterilization by 72 h (Fig. 4A). Incubation of M. abscessus with 1/4× or 1/8× MIC of CLR and TGC allowed the growth of M. abscessus (Fig. 4B and C). In both cases, supplementation with RFB at 1/4× MIC was bacteriostatic, while increasing RFB to 1× MIC caused a large reduction in CFU (Fig. 4B and C). These data confirmed the 3D checkerboard assays showing that the triple combination of RFB, CLR, and TGC has potent inhibitory effects on M. abscessus and indicated that this activity can be bactericidal.

To test the synergistic effects of RFB on M. abscessus viability at concentrations of CLR and TGC achieved in the lung epithelial lining fluid (8, 21), M. abscessus was incubated in broth containing 20 mg/liter CLR (50× MIC) and 0.4 mg/liter TGC (1/2× MIC), along with various concentrations of RFB. Lung attainable concentrations of CLR and TGC caused reductions in M. abscessus CFU (Fig. 4D) superior to those found at 1× MICs of CLR and TGC (Fig. 4A). The addition of RFB further enhanced the reduction of M. abscessus CFU. Addition of RFB at concentrations as low as 1/4× MIC reduced CFU to below the limit of detection by 96 h (Fig. 4D). Addition of RFB at 1/8× or 1/16× MIC also reduced M. abscessus CFU compared to treatment with the CLR and TGC combination alone but did not sterilize. These results demonstrate that the addition of RFB to clinically relevant concentrations of CLR and TGC mixtures synergistically increases the growth-inhibitory effects and is bactericidal.

## DISCUSSION

Routine treatment of many bacterial infections requires administration of multiple antibiotics to enhance killing or minimize the development of resistance. For example, the use of beta-lactam antibiotics with gentamicin results in synergistic bactericidal activity against Enterococcus spp. and improvement of patient outcomes (22). In a screen for antibiotics active against M. abscessus, we identified RFB and showed that it was synergistic with CLR and TGC in both growth and time-kill analyses. We did not observe antagonistic interactions of RFB with any antibiotic used to treat M. abscessus infections. We suggest that inclusion of RFB as a partner in combined anti-M. abscessus therapies should be further investigated.

Current treatment of M. abscessus infections consists of CLR coadministered with AMK and either FOX or IPM. However, meta-analyses show that treatment outcomes are extremely poor with clearance rates of only 41% when administered along with adjunct surgery (35% without) (3). This was corroborated by another meta-analysis that found only 23% of patients had good treatment outcomes (23). A likely explanation for this is that CLR induces expression of *whiB7*, a global regulator of intrinsic resistance genes, which causes upregulation of *erm*(41) (which confers resistance to macrolides) and *eis2* (which confers resistance to AMK) (7, 24). Attempts to find drugs that positively interact with existing M. abscessus therapies have revealed synergism of TGC and CLR in multiple strains (19, 20), and synergism of clofazimine and AMK (25–27). In a Drosophila infection model, TGC was also synergistic with linezolid for prolonging life and reducing colonization (28). IPM is often synergistic with CLR or levofloxacin (29). However, one study found 96% of strains to be levofloxacin resistant (30), making the practical utility of the interaction doubtful.

In screening the Sweet compound library, we found that RFB was active against M. abscessus (Table 1), confirming a recent independent study (11). These studies raise the question of why RFB is the only rifamycin derivative that is active against M. abscessus. It presumably reflects either better affinity for the M. abscessus RNA polymerase or poor activity of an intrinsic resistance system. M. abscessus contains an ADP-ribosyltransferase (MAB_0591) that is responsible for inactivation and resistance to rifamycin, rifaximin, and rifapentine (10). Comparative studies of RFB binding to M. abscessus RNAP or the specificities of MAB_0591 ADP-ribosyltransferase have not been done. RFB pharmacokinetics are not well established, but one analysis found that it has a very good volume of distribution (9.3 liters/kg) and a very long half-life (45 h), suggesting lung concentrations should be constant throughout the day (18). The serum *C*_max_ of RFB was only 0.46 mg/liter at 2.3 h (*T*_max_); however, like other rifampin analogues, RFB accumulates in the lungs with a 6- to 7-fold increase 12 h posttreatment (18). This suggests the lung concentration at 12 h should be 2.4 to 2.8 mg/liter with an extrapolated *C*_min_ at 24 h of 2 to 2.3 mg/liter. Unfortunately, this is a concentration lower than the RFB MIC (Table 1) (11). Utilization of synergistic drug combinations could reduce the RFB MIC, making it therapeutically relevant.

While rifamycins are synergistic with carbapenems and cephalosporins against M. abscessus (31) and M. tuberculosis (32), RFB synergies have not been reported. Unfortunately, our data showed that IPM and FOX, the only carbapenem and cephalosporin indicated for use with M. abscessus, did not exhibit synergy with RFB (data not shown). Analysis of the interaction of RFB and macrolides (CLR, AZM, and ROX) revealed synergistic inhibitory effects on M. abscessus growth (Tables 2 and 3). CLR, a foundation of M. abscessus therapy, is problematic because its primary effect at therapeutic concentrations is bacteriostatic and not bactericidal, and it also induces expression of resistance controlled by *whiB7* (7, 24). Rifampin (and by analogy RFB) has concentration dependent bactericidal activity against M. tuberculosis (33), and any synergistic drug interactions that increase the *C*_max_/MIC ratio may accelerate the rates of killing. Our data showed that the addition of macrolides CLR, AZM, or ROX to RFB (each partner at 1× MIC) caused a 3- to 4-log loss in viability over 96 h (Fig. 1C; see also Fig. S2 in the supplemental material). This was even more pronounced at higher CLR concentrations (50× MIC) likely encountered in the lungs. In these experiments, compared to CLR alone, CLR in combination with 6 or 3 mg/liter RFB (1× and 1/2× MIC) caused a >5-log decrease in CFU (below the limit of detection), and 1.6 to 0.4 mg/liter (1/4× to 1/16× MIC) RFB resulted in a 2- to 3-log reduction in CFU (Fig. 1E). Similarly, TGC in combination with RFB caused a synergistic arrest of growth (Table 2) and a reduction in viability (CFU analysis, Fig. 2).

Initial studies have explored the use of tigecycline therapies for M. abscessus infections. Although TGC has a large volume of distribution and intracellular accumulation (34, 35), it has limited distribution to lung tissue. Its *C*_max_ (0.4 to 0.8 mg/liter) in the epithelial lining fluid (21, 36) was determined to be lower than its M. abscessus MIC in one study (0.5 to 2 mg/liter) (37) and only slightly higher than the MIC (0.25 mg/liter) in another study (38). Although TGC has shown some promising results for salvage therapy (55% of cystic fibrosis patients with pulmonary infection showed improvement), it is often reserved for other infections and its place in M. abscessus therapy has not been established (39). Previous assays of M. abscessus demonstrated that CLR and TGC have synergistic effects on growth inhibition, but our studies suggest this activity is not bactericidal (Fig. 3).

A major disadvantage of M. abscessus treatment is the requirement for prolonged therapy (6 to 12 months), which can allow emergence of antibiotic resistance and have adverse effects on patients (2). The bactericidal activity of RFB in combination with either CLR and/or TGC could improve outcomes or reduce the time needed for treatment. Our studies using 3D checkerboards showed that a triple combination of RFB, CLR, and TGC displayed synergistic effects. The triple combination had an FICI of 0.375 against M. abscessus ATCC 19977 and FICIs of 0.375 to 0.625 for six independent clinical strains (Table 4). Triple combination therapy could reduce the MIC of RFB 8-fold, to 0.8 mg/liter (Table 4), allowing it to become active at concentrations achieved in the lungs (2 to 2.3 mg/liter) (18). Importantly, the triple combination had bactericidal effects (Fig. 4). When present along with clinically relevant concentrations of CLR and TGC, RFB reduced CFU to below the limit of detection at ≥1.6 mg/liter (Fig. 4D). In addition to this pharmacokinetic data, pharmacodynamic studies have shown that coadministration of CLR and RFB increases plasma concentrations of RFB and increases the concentrations of CLR's active metabolite (18). During respiratory infections, M. abscessus resides in intracellular environments that are accessible by RFB, TGC, and CLR (40–43). These antibiotics are all able to inhibit intracellular bacteria (40–43), suggesting that their combined activity is likely to be synergistic against M. abscessus within this niche.

The utilization of rifampin analogues in M. abscessus has not been investigated due to their poor *in vitro* activities. However, RFB does have favorable pharmacokinetic properties, including a good volume of distribution, accumulation in lung tissue (6× to 7× serum levels), and a long half-life (45 h) (18). Although the MIC of RFB alone is below what may be achievable in lung tissues, in combination with CLR and TGC, sterilizing concentrations of RFB are achievable in the lungs. Given the poor outcomes of M. abscessus treatment in clinical settings, better combination therapy is needed both to avoid antagonistic interactions and to favor synergic interactions. We suggest that combinations including RFB should be further investigated for treatment of M. abscessus pulmonary infections.

## MATERIALS AND METHODS

### Bacterial strains.

M. abscessus strain ATCC 19977 was purchased from the American Type Culture Collection, and clinical M. abscessus strains were obtained from Patrick Tang at the British Columbia Centre for Disease Control. The M. abscessus strains used in these studies all had a rough-colony phenotype. All precultures were grown in Mueller Hinton II (MHII) medium supplemented with 0.05% tyloxapol at 37°C in rolling test tubes to a final optical density at 600 nm (OD_600_) of 2 to 5. They were then diluted into unsupplemented MHII medium for *in vitro* testing of antibiotic sensitivity.

### MIC determination.

Precultures were diluted to an OD_600_ of 0.005 in MHII medium, and 100 μl was added to 100 μl of MHII medium containing serial 2-fold dilutions of antibiotics in 96-well plates (Costar, catalog no. 3370). Plates were then incubated for 48 h, followed by the addition of 30 μl of resazurin-water (10 mg/100 ml). Plates were incubated for an additional 24 h, and growth was recorded as conversion of color from blue to pink.

### FICI determination.

The FICI was determined in 96-well plates in a checkerboard format using a resazurin assay (13). The FICI for each compound was calculated as follows. FIC_A_, the fractional inhibitory concentration of compound A, is the MIC of compound A in the presence of compound B/MIC of compound A alone. The FIC_B_ for compound B was similarly calculated. The FICI was calculated as FIC_A_ plus FIC_B_. 3D checkerboards were developed to measure the effect of adding another antibiotic in a triple combination (44). The FICI was analyzed as previously described (15, 45, 46). In both 2D and 3D checkerboard analyses, drug interactions were defined as synergistic when they had FICI values of ≤0.75 (14–17). The MICs of RFB in combination with ranges of CLR and TGC concentrations were plotted in three dimensions (15, 17) using SURFER 15 software (Golden Software, Inc., Golden, CO).

### CFU analysis.

Precultures were diluted to an OD_600_ of 0.005 in 3 ml of MHII medium in test tubes. After the cultures entered exponential growth phase (OD_600_ 0.7 to 1.5), they were diluted to an OD_600_ of 0.005, and 3 ml was added to test tubes with appropriate concentrations of RFB, CLR, AZM, ROX, or TGC. At specified times, 100 μl of culture was removed from each tube, and serial 10-fold dilutions were performed. Then, 10 μl of each dilution was spotted onto MHII agar plates, which were incubated at 37°C for 5 days, and the colonies were counted.

## Supplementary Material

Supplemental file 1
