# Supplementary material for: Rifabutin Acts in Synergy and Is Bactericidal with Frontline Mycobacterium abscessus Antibiotics Clarithromycin and Tigecycline, Suggesting a Potent Treatment Combination
Source: Antimicrob Agents Chemother. 2018 Jul 27;62(8):e00283-18. doi: 10.1128/AAC.00283-18 (PMC6105836; doi:10.1128/AAC.00283-18)
Supplement: Supplemental file 1 [file zac008187308s1.pdf]

SUPPLEMENTARY MATERIAL

Rifabutin acts in synergy and is bactericidal with frontline *Mycobacterium abscessus* antibiotics clarithromycin and tigecycline, suggesting a potent treatment combination

Mark Pryjma, Jan Burian, and Charles J. Thompson*
Department of Microbiology and Immunology and the Centre for Tuberculosis Research, University of British Columbia, Vancouver, Canada, V6T 1Z3
*To whom correspondence should be addressed:
Tel: 1-604-822-2501
Email: charles.thompson@ubc.ca

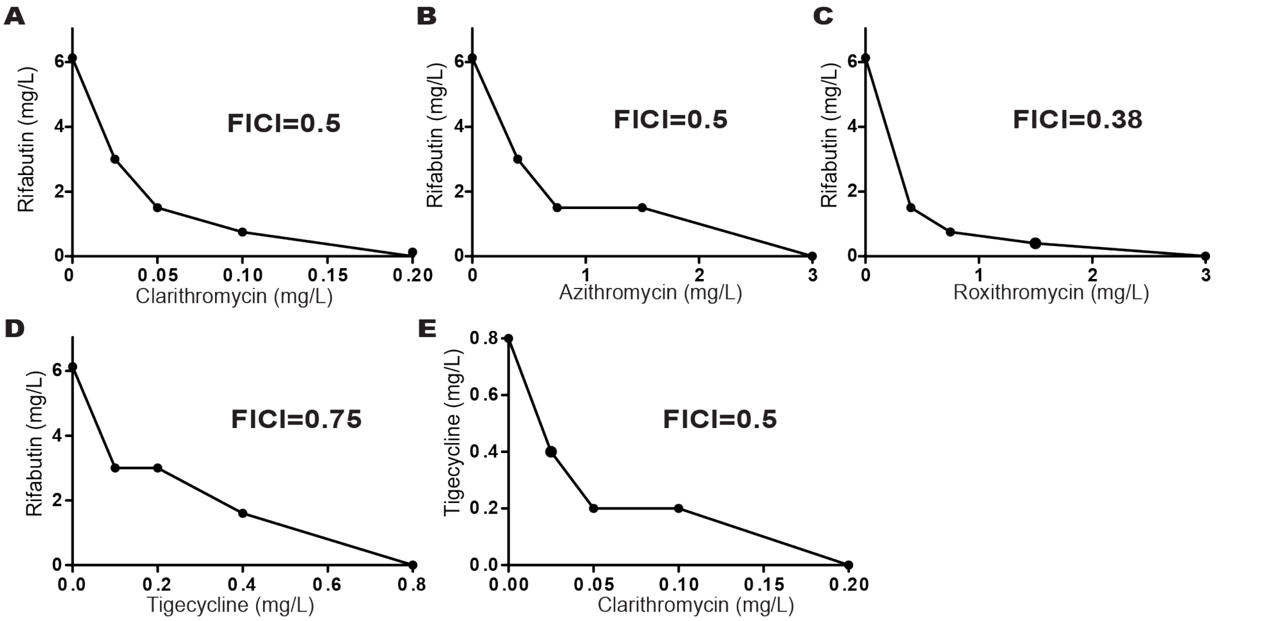

Figure S1. *Mycobacterium abscessus* checkerboard plots of rifabutin in combination with various antibiotics. *MAB* ATCC19977 MICs to rifabutin were measured in the presence of sub-MIC concentrations of **A)** clarithromycin, **B)** azithromycin, **C)** roxithromycin or **D)** tigecycline. Alternatively, **E)** the tigecycline MIC was measured in the presence of sub-MIC concentrations of clarithromycin. Concave curves represent positive interactions between two antibiotics approaching synergy. FICI values for antibiotic combinations are shown. FICI ≤0.75 represents synergy.

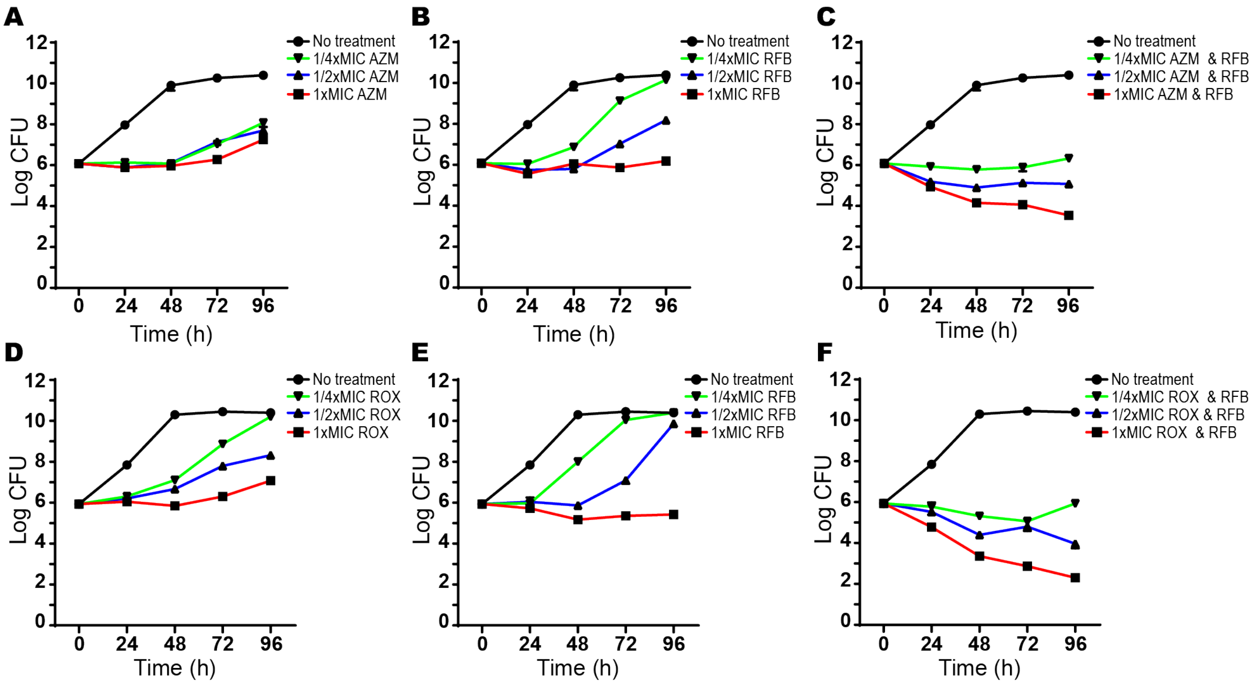

Figure S2. Effect of rifabutin and azithromycin or roxithromycin combinations on *Mycobacterium abscessus* viability. *MAB* ATCC19977 cultures at OD600=0.005 were exposed to **A**) azithromycin at 1x (3.1 mg/L), 1/2x or 1/4xMIC, **B&E)** Rifabutin at 1x (6.3 mg/L), 1/2x or 1/4xMIC, **C)** a combination of azithromycin and rifabutin at 1x, 1/2x, and 1/4xMIC of each antibiotic, **D)** roxithromycin at 1x (3.1 mg/L), 1/2x or 1/4xMIC, or **F)** roxithromycin and rifabutin at 1x, 1/2x or 1/4xMIC of each antibiotic. CFUs were determined at 24 h intervals post antibiotic addition. The dashed line represents the limit of detection. Data points are the means of three replicates with standard deviation presented as error bars.

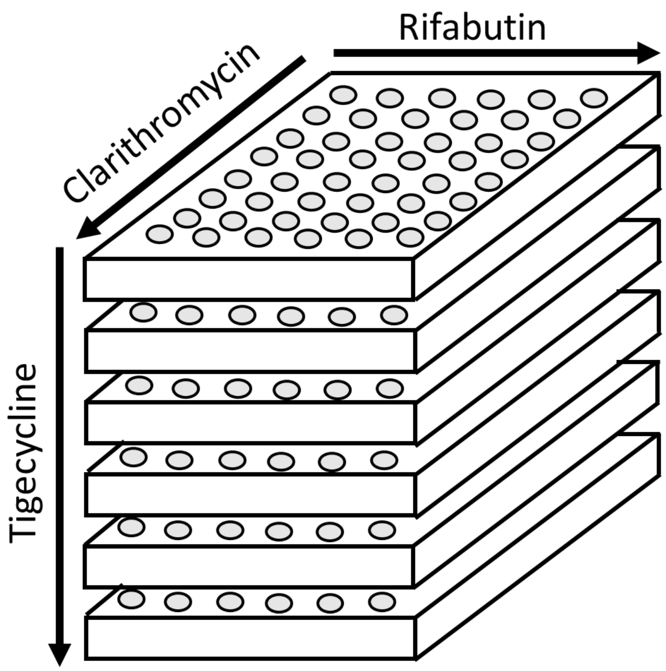

Figure S3. **Schematic representation of 3D checkerboard.** Arrows describe descending concentrations of the respective antibiotic along the axis indicated.

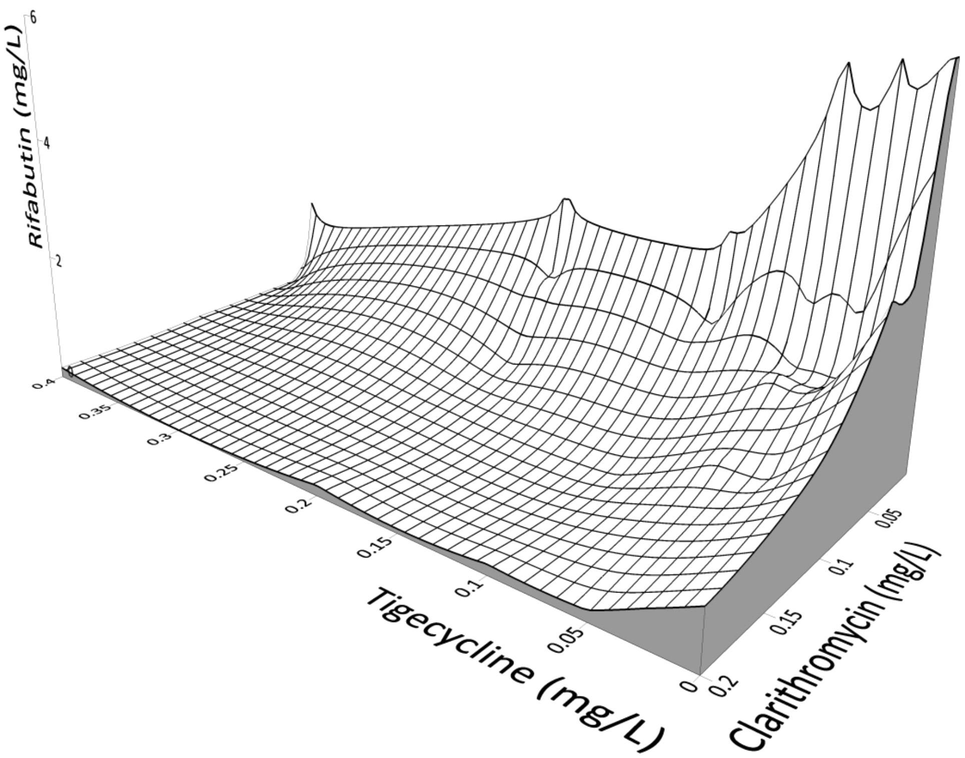

Figure S4. Isobologram showing interaction of clarithromycin, tigecycline and rifabutin against *Mycobacterium abscessus*. Concave areas represent synergistic drug interactions.
